# Impact of physical activity on preeclampsia and angiogenic markers in the Finnish Genetics of Pre-eclampsia Consortium (FINNPEC) cohort

**DOI:** 10.1080/07853890.2024.2325480

**Published:** 2024-03-11

**Authors:** Noora Jaatinen, Eeva Ekholm, FINNPEC, Hannele Laivuori, Tiina Jääskeläinen

**Affiliations:** a Department of Obstetrics and Gynecology, Turku University Central Hospital and University of Turku, Turku, Finland; b Department of Obstetrics and Gynecology, University of Helsinki and Helsinki University Hospital, Helsinki, Finland; c Medical and Clinical Genetics, University of Helsinki and Helsinki University Hospital, Helsinki, Finland; d Institute for Molecular Medicine Finland (FIMM), Helsinki Institute of Life Science, University of Helsinki, Helsinki, Finland; e Department of Obstetrics and Gynecology, Faculty of Medicine and Health Technology, Center for Child, Adolescent, and Maternal Health Research, Tampere University Hospital and University of Tampere, Tampere, Finland; f Department of Food and Nutrition, University of Helsinki, Helsinki, Finland

**Keywords:** Angiogenic markers, preeclampsia, physical activity, pregnancy, pregnancy complication

## Abstract

**Introduction:**

Effect of physical activity in pregnancy on preeclampsia (PE) and angiogenic markers is not well understood. We studied the association of physical activity and PE in a case-control setting and assessed whether exercise in PE and non-PE women associate with maternal serum concentrations of soluble fms-like tyrosine kinase 1 (s-Flt-1), placental growth factor (PlGF) and soluble endoglin (sEng) and sFlt-1/PlGF ratio in the Finnish Genetics of Pre-eclampsia Consortium (FINNPEC) cohort.

**Materials and methods:**

Participants completed a questionnaire on their background information and serum samples were collected from a subset. Questionnaire data on physical activity were available from 708 PE women and 724 non-PE women. Both first trimester serum samples and questionnaire data on physical activity were available from 160 PE women and 160 non-PE women, and second/third trimester serum samples and questionnaire data on physical activity were available from 139 PE women and 47 non-PE women. The PE and non-PE women were divided into categories of physically active (exercise 2 − 3 times/week or more) and physically inactive (exercise less than 2 − 3 times/week).

**Results:**

A total of 43.4% of the PE women and 42.4% of the non-PE women were categorized as physically active. There were no differences in physical activity and exercise habits between the groups. The physically active women were more often nulliparous and non-smokers and had a lower body mass index. There were no differences in the concentrations of angiogenic markers (sFlt-1, PlGF and sEng and sFlt-1/PlGF ratio) between the groups who exercised more or less than 2 − 3 times/week.

**Conclusions:**

In the FINNPEC study cohort, there was no association between physical activity and PE and no associations of physical activity in pregnant women with and without PE with maternal serum concentrations of sFlt-1, PlGF and sEng and sFlt-1/PlGF ratio.

## KEY MESSAGES

This is the first study to investigate the association of physical activity in pregnancy with concentrations of angiogenic markers while comparing pregnant women with and without preeclampsia.

There were no differences in the physical activity and exercise habits in pregnancy between women with and without preeclampsia in the FINNPEC cohort.

Physical activity of pregnant women with or without preeclampsia did not associate with the concentrations of angiogenic markers (sFlt-1, PlGF and sEng and sFlt-1/PlGF ratio).

## 1. Introduction

Preeclampsia (PE) is a complex pregnancy-specific ­disorder that is characterized by new-onset hypertension and proteinuria after 20 weeks of gestation or new-onset signs of other maternal end-organ dysfunction or uteroplacental dysfunction in the absence of proteinuria [1]. PE affects 3–5% of pregnancies and is one of the main causes of maternal, fetal and neonatal morbidity and mortality [2]. The etiology of PE is largely unknown, and its prevention remains a challenge. Reduced placental perfusion and inflammation associated with oxidative stress and endothelial dysfunction are considered as central features in the pathogenesis of PE [3].

Exercise during pregnancy has been suggested as protection from PE [4–7], but the data are conflicting [8–10]. The 2021 International Society for the Study of Hypertension in Pregnancy stated that exercise in pregnancy is recommended for all women without contraindications for a reduction of the likelihood of PE [1]. To achieve these reductions, women should undertake at least 140 min per week of moderate-intensity exercise [1]. The mechanisms by which exercise reduces the risk of PE are not well understood, but it has been suggested that exercise training promotes placental growth and vascular development, reduces oxidative stress and improves endothelial function as well as immune and inflammatory responses [11].

Angiogenic markers are involved in the pathogenesis of PE [12]. Circulating maternal serum levels of soluble fms-like tyrosine kinase 1 (s-Flt-1) and soluble endoglin (sEng) and sFlt-1/PlGF ratio are increased and levels of placental growth factor (PIGF) are decreased in PE as well as in pregnant women weeks prior to established PE [13, 14].

There are limited data on angiogenic markers and exercise in pregnancy. In one small study, regular exercise during pregnancy was associated with higher serum PlGF and lower sFlt-1 and sEng concentrations in late non-complicated pregnancy [15]. This kind of pro-angiogenic serum profile is hypothesized to contribute to the reduced risk of PE among physically active women [15]. Exercise training during pregnancy was shown to decrease s-Flt-1 levels in a rat model of hypertension during PE [16] and a rat model of PE superimposed on chronic hypertension [17]. Human studies on the association of exercise in pregnancy, PE and angiogenic markers are lacking.

Our aims were first to study the association between physical activity and PE in a case-control setting and second to assess whether exercise in pregnant women with and without PE associate with maternal serum concentrations of sFlt-1, PlGF and sEng and sFlt-1/PlGF ratio in the Finnish Genetics of Pre-eclampsia Consortium (FINNPEC) cohort.

## 2. Material and methods

### 2.1. Study design

The data consisted of the prospective arm of the FINNPEC cohort. FINNPEC, a cross-sectional case-control multicenter study, was established to set up a nationwide clinical and DNA database of pregnant women with and without PE, their partners and infants. The cohort was established in order to identify genetic risk factors for PE. The details of the study design, methods and procedures have been previously published [18–20].

### 2.2. Study subjects

A total of 923 pregnant women with PE and 1009 controls (non-PE) were recruited for the study from 2008 to 2011. The inclusion criteria were age above 18 years, a singleton pregnancy and the ability to provide informed consent based on information in Finnish or Swedish. PE was defined as hypertension and proteinuria occurring after 20 weeks of gestation as based on the American College of Obstetricians and Gynecologists 2002 criteria [21]. Hypertension was defined as systolic blood pressure ≥ 140 mmHg and/or diastolic blood pressure ≥ 90 mmHg. Proteinuria was defined as the urinary excretion of ≥ 0.3 g protein in a 24-h specimen, 0.3 g/l or two ≥ 1+ readings on a dipstick in a random urine sample with no evidence of a urinary tract infection. Each diagnosis was independently verified from medical records by a research nurse and research physician. The control group consisted of healthy women with uncomplicated pregnancies and women with pregnancy complications excluding PE, such as gestational diabetes, gestational hypertension, proteinuria without high blood pressure, placental insufficiency, PE in a previous pregnancy, fetal death and small for gestational age fetuses.

All participants provided written informed consent, and the FINNPEC study protocol was approved by the coordinating Ethics Committee of the Hospital District of Helsinki and Uusimaa (149/EO/2007).

The participants completed a detailed questionnaire on their background information that included data on physical activity during pregnancy. Information on age, pre-pregnancy weight and height, obstetric history, medical history, pregnancy complications, pregnancy outcome, blood pressure, delivery and newborn were obtained from hospital records and maternity cards. Data on smoking were collected from maternity cards and complemented by the background information questionnaires.

The questionnaire included eight questions about physical activity and exercise habits during pregnancy. The questions defined how much time was spent exercising, how strenuous the exercise was and if it was performed during work or leisure time. By combining the answers to different questions, two variables were set to divide the women into two categories according to their physical activity: physically active and inactive. A new variable (How often do you exercise?) was created from the question ‘How often do you exercise?’ with answer options: never, less than once a month, about once a week, 2–3 times a week, 4–5 times a week or approximately every day. The new variable divided women into categories of physically active (exercise 2 − 3 times/week or more) and physically inactive (exercise less than 2 − 3 times/week). We also created another variable (Do you exercise ≥ 2–3 times/week, ≥ 30 min at a time with at least moderate intensity? [Yes/No]) by combining data from three questions (How often do you exercise? How long does an average free-time exercise session last? Is your free-time exercise about as strenuous as alternating between walking and light running?). These categorizations were based on the recommendations of the Finnish Current Care Guidelines for exercise during pregnancy (exercise training at least 150 min/week divided among at least 3 days/week) [22].

### 2.3. Serum samples and angiogenic markers

First and second/third trimester serum samples were available from women who received care in the Hospital District of Helsinki and Uusimaa. First trimester serum samples were obtained from the first trimester biochemical screening for fetal chromosomal abnormalities (range 9–15 weeks of gestation), and serum samples from the second/third trimesters (range 20–42 weeks of gestation) were collected at hospitals according to the study protocol.

Maternal serum sFlt-1 and PlGF concentrations were measured using sFlt-1 and PlGF electro-chemiluminescence immunoassays (ECLIA; Roche Diagnostics GmbH, Mannheim, Germany) on a cobas e 601 analyzer (Hitachi High Technology Co, Tokyo, Japan). Serum concentration of endoglin (CD105) was measured using a human Quantikine Endoglin ELISA kit (R&D Systems, UK) according to the manufacturer’s instructions.

### 2.4. Statistical analysis

Statistical tests were performed using SPSS Statistics 25.0 (IBM Corp., Armonk, NY, USA). The physical activity of the PE and non-PE women was compared. The maternal and perinatal characteristics of the PE and non-PE women were compared according to physical activity separately.

The normality of the variable distributions was verified graphically and with a Kolmogorov–Smirnov-test. The statistical analyses of the continuous variables were performed using two-sample t-tests for the normal distributions and Mann–Whitney U-tests for the skewed distributions. The categorical variable comparisons between the groups were performed with Chi-square tests. *P* values of < 0.05 were considered as statistically significant.

The first and second/third trimester serum concentrations of angiogenic markers in PE and non-PE women divided according to physical activity were compared. Logarithmic transformation was used when appropriate. Each biomarker was ln-transformed to correct for right-skewness, and estimated means were back-transformed as mean estimates/model-based means and 95% confidence intervals for purposes of presentation. Comparisons between groups were analyzed with a two-way analysis of variance (ANOVA). Selected co-variables (parity, maternal age, smoking status, body mass index [BMI]) were included in the models as covariates. The normality of the variable distributions was verified graphically and with a Kolmogorov–Smirnov-test.

## 3. Results

### 3.1. Physical activity

Information on the study participants’ physical activity is shown in Table 1. Questionnaire data on physical activity were available from 708 PE women and 724 non-PE women. A total of 43.4% of PE women and 42.4% of non-PE women exercised ≥ 2–3 times/week, ≥ 30 min at a time with at least moderate intensity and were thus categorized as physically active. The women with PE estimated their physical fitness to be poorer than did the non-PE women; however, there were no clear differences in physical activity and exercise habits between the groups. The women with PE spent less time daily doing household work, yard work and/or gardening than the non-PE women.

**Table 1.** Physical activity of the FINNPEC women.

	PE (*n* = 708)		Non-PE (*n* = 724)		
	*N*	%	*N*	%	*p* Value
Physical fitness status, own estimate	695		715		**0.001**
Good	298	42.9	373	52.2	
Average	286	41.2	263	36.8	
Poor	111	16.0	79	11.0	
Time spent physically active^a^ daily when traveling to workplace	571		560		0.169
<15 min	272	47.6	236	42.1	
15–30 min	146	25.6	154	27.5	
>30 min	153	26.8	170	30.4	
How physically strenuous is your job?	569		544		0.463
Light (mostly sitting)	276	48.5	264	48.5	
Somewhat light	122	21.4	131	24.1	
Somewhat strenuous or strenuous	171	30.1	149	27.4	
How often do you exercise?	685		709		0.866
1–2 times/month or less	135	19.7	137	19.3	
1 time/week	136	19.9	152	21.4	
2–3 times/week	264	38.5	274	38.6	
4–5 times/week or more	150	21.9	146	20.6	
How strenuous is your exercise?	672		699		0.452
Similar to walking	255	37.9	236	33.8	
Similar to walking and jogging in turns	247	36.8	274	39.2	
Similar to jogging	136	20.2	150	21.5	
Similar to running	34	5.1	39	5.6	
The length of an exercise session	681		701		0.566
<30 min	50	7.3	46	6.6	
30–60 min	330	48.5	359	51.2	
>60 min	301	44.2	296	42.2	
Time spent otherwise physically active daily^b^	691		718		**0.036**
<30 min	181	26.2	195	27.2	
30–60 min	304	44.0	278	38.7	
1–2 h	164	23.7	175	24.4	
≥2 h	42	6.1	70	9.7	
Physically active vs inactive					
How often do you exercise?	685		709		0.648
2–3 times/week or more	414	60.4	420	59.2	
Less than 2–3 times/week	271	39.6	289	40.8	
Do you exercise ≥2–3 times/week, ≥30 min at a time with at least moderate intensity^c^?	647		684		0.703
Yes	281	43.4	290	42.4	
No	366	56.6	394	57.6	

^a^Walking, running, cycling or cross-country skiing. ^b^Including household work, yard work and gardening. ^c^Similar to walking and jogging in turns. Bold text shows *p* values < 0.05. () Number of available information sources if not from all.

### 3.2. Maternal and perinatal characteristics according to physical activity

The maternal and perinatal characteristics of the PE and non-PE women according to physical activity are presented in Table 2. Among both the PE and non-PE women, those who were physically active were more often nulliparous, non-smokers before and during pregnancy and had a lower BMI. In the non-PE group, the physically active women were younger and had fewer instances of gestational diabetes. There were no differences in any other characteristics between the physically active and inactive women, including blood pressure levels, modes of delivery, gestational weeks at delivery, birth weights and small for gestational age fetuses (birth weights below −2.0 standard deviation [SD] units, according to Finnish standards).

**Table 2.** Maternal and perinatal characteristics according to physical activity category in the FINNPEC.

	PE			Non-PE		
	Physically active (*n* = 414)	Physically inactive (*n* = 271)	*p*	Physically active (*n* = 420)	Physically inactive (*n* = 289)	*p*
Age at delivery (years)	30.2 ± 5.3 (*n* = 413)	29.4 ± 5.9	0.053	29.5 ± 4.8 (*n* = 419)	30.3 ± 5.3 (*n* = 287)	**0.032**
Nulliparous, *n* (%)	333 (80.6%) (*n* = 413)	186 (68.6%)	**<0.001**	270 (64.4%) (*n* = 419)	126 (43.9%) (*n* = 287)	**<0.001**
BMI, kg/m^2^ (self-reported, pre-pregnancy)	23.4 (16.2 − 45.9)^a^ (*n* = 413)	24.8 (17.0 − 47.3)^a^	**<0.001**	22.8 (17.1 − 42.9)^a^ (*n* = 419)	23.8 (17.0 − 47.4)^a^ (*n* = 287)	**0.002**
Smoking before pregnancy	104 (25.4%) (*n* = 409)	101 (37.3%)	**0.001**	105 (25.5%) (*n* = 412)	101 (35.4%) (*n* = 285)	**0.005**
Smoking during pregnancy	27 (6.5%) (*n* = 413)	36 (13.3%)	**0.003**	35 (8.3%)	48 (16.7%) (*n* = 287)	**0.001**
Highest systolic blood pressure (mmHg)	165 (118 − 235) (*n* = 413)	163 (129 − 239)	0.066	128 (100 − 200) (*n* = 419)	127 (100 − 214) (*n* = 287)	0.419
Highest diastolic blood pressure (mmHg)	109 (88 − 173) (*n* = 413)	108 (91 − 130)	0.177	84 (62 − 134) (*n* = 419)	84 (65 − 123) (*n* = 287)	0.971
Highest systolic blood pressure at first antenatal visit (mmHg)	124 (90 − 170) (*n* = 405)	124 (94 − 194) (*n* = 262)	0.985	119 (87 − 190) (*n* = 404)	118 (90 − 160) (*n* = 282)	0.308
Highest diastolic blood pressure at first antenatal visit (mmHg)	78 (52 − 109) (*n* = 405)	78 (52 − 116) (*n* = 262)	0.889	73 (50 − 124) (*n* = 404)	73 (50 − 106) (*n* = 282)	0.827
Early onset PE^b^	91 (22.0%)	62 (22.9%)	0.783	–	–	–
Chronic hypertension^c^	77 (18.6%) (*n* = 413)	42 (15.5%)	0.288	21 (5.0%) (*n* = 419)	22 (7.7%) (*n* = 287)	0.148
Gestational hypertension^d^	–	–	–	46 (11.0%) (*n* = 419)	20 (7.0%) (*n* = 287)	0.072
Pre-gestational diabetes	8 (1.9%) (*n* = 413)	12 (4.4%)	0.059	4 (1.0%) (*n* = 419)	2 (0.7%) (*n* = 287)	0.714
Type 1 diabetes	7 (1.7%)	10 (3.7%)		1 (0.2%)	3 (1.0%)	
Type 2 diabetes	1 (0.2%)	2 (0.7%)		1 (0.2%)	1 (0.3%)	
Gestational diabetes	61 (14.8%) (*n* = 413)	54 (19.9%)	0.078	31 (7.4%) (*n* = 419)	35 (12.2%) (*n* = 287)	**0.032**
Mode of delivery	(*n* = 413)		0.339		(*n* = 288)	0.242
Vaginal	244 (59.1%)	170 (62.7)		362 (86.2%)	239 (83.0%)	
Cesarean section	169 (40.9%)	101 (37.3)		58 (13.8%)	49 (17.0%)	
Gestational weeks at delivery	37 (24 − 42) (*n* = 413)	38 (24 − 41)	0.474	40 (26 − 42)	40 (23 − 43) (*n* = 288)	0.825
Birth weight, g	2880 (310 − 4660)^a^ (*n* = 413)	2960 (700 − 4840)^a^	0.235	3570 (460 − 5010)^a^	3548 (330 − 5350)^a^ (*n* = 288)	0.763
Relative birth weight (SD)	−0.97 ± 1.22 (*n* = 413)	−0.81 ± 1.22	0.910	−0.15 ± 1.1	−0.09 ± 1.1 (*n* = 287)	0.693
SGA	81 (19.6%) (*n* = 413)	46 (17.0%)	0.385	22 (5.3%) (*n* = 419)	14 (4.9%) (*n* = 287)	0.825
Placental insufficiency	37 (9.0%) (*n* = 413)	26 (9.6%)	0.779	13 (3.1%) (*n* = 419)	10 (3.5%) (*n* = 287)	0.779
Preterm delivery (< 37 gwks)	127 (30.8%) (*n* = 413)	79 (29.8%)	0.656	20 (4.8%)	14 (4.8%)	0.960

Data are presented as mean ± SD or percentages. ^a^Median (range). ^b^PE was identified as early onset if diagnosed before 34^+0^ gwks. ^c^Systolic blood pressure ≥ 140 mmHg and/or diastolic blood pressure ≥ 90 mmHg detected before 20 weeks of gestation. ^d^Blood pressure ≥ 140/90, no proteinuria. Bold text shows *p* values < 0.05. () Number of available information sources if not from all.

### 3.3. Angiogenic markers in PE and non-PE women according to physical activity

Both first trimester serum samples and the questionnaire data on physical activity were available from 160 PE women and 160 non-PE women, and second/third trimester serum samples and questionnaire data on physical activity were available from 139 PE women and 47 non-PE women. The maternal and perinatal characteristics of the subsets of PE and non-PE women with first and second/third trimester serum samples of angiogenic markers are presented in Supplementary Table 1. In these subsets the PE women had higher blood pressure, higher rates of thrombocytopenia, delivered earlier and had infants of lower birth weight than the non-PE women. The maternal and perinatal characteristics of the whole cohort of the FINNPEC women, with available questionnaire data including 708 PE women and 724 non-PE women, are presented in Supplementary Table 2. The PE women in the whole cohort had higher BMI and higher rates of gestational diabetes and placental insufficiency compared to non-PE women. In the subsets of women with serum samples, there were no similar differences between the groups.

The first and second/third trimester serum concentrations of angiogenic markers in the PE and non-PE women according physical activity are presented in Table 3. There were no differences in the concentrations of angiogenic markers between the groups who exercised more or less than 2 − 3 times/week. Physical activity was not associated with the concentrations of angiogenic markers. The same analyses were done using the variable ‘Do you exercise ≥ 2–3 times/week, ≥ 30 min at a time with at least moderate intensity?’ with similar results (data not shown).

**Table 3.** Concentrations of angiogenic markers in PE and non-PE women according to physical activity category, mean estimate/model-based mean (95% CI).

	PE		Non-PE		*p* Value			
	Physically active^a^	Physically inactive^b^	Physically active^a^	Physically inactive^b^	Active vs inactive	Active vs inactive^c^	Group * exercise interaction	Group * exercise interaction^c^
s-Flt1 (pg/ml)								
I trimester	1311.6 (1208.3 − 1422.3) (*n* = 104)	1280.49 (1146 − 1430.8) (*n* = 56)	1418 (1301.1 − 1546.9) (*n* = 94)	1399.7 (1264 − 1550) (*n* = 66)	0.707	0.517	0.919	0.980
III trimester	10938 (9623.9 − 12431.6) (*n* = 87)	9348.8 (7918.8 − 11025.9) (*n* = 52)	4505.3 (3565.3 − 5693) (*n* = 26)	4514.3 (3480.7 − 5860.6) (*n* = 21)	0.454	0.343	0.441	0.143
s-PlGF (pg/ml)								
I trimester	31.7 (29.3 − 34.3) (*n* = 104)	28.5 (25.6 − 31.8) (*n* = 55)	41.6 (38.3 − 45.3) (*n* = 94)	41.0 (37.1 − 45.2) (*n* = 66)	0.202	0.284	0.347	0.420
III trimester	78.7 (68.2 − 90.9) (*n* = 87)	88.9 (73.7 − 107) (*n* = 52)	150.4 (115.5 − 195.6) (*n* = 26)	170.5 (127.1 − 228.6) (*n* = 21)	0.291	0.285	0.981	0.918
s-Eng (ng/ml)								
I trimester	6.1 (5.8 − 6.46) (*n* = 103)	5.7 (5.3 − 6.1) (*n* = 56)	5.7 (5.4 − 6.0) (*n* = 94)	5.4 (5.1 − 5.8) (*n* = 66)	0.055	0.419	0.726	0.802
III trimester	43.6 (38.2 − 50.0) (*n* = 87)	37.8 (31.8 − 44.9) (*n* = 52)	19.5 (15.3 − 24.9) (*n* = 26)	14.9 (11.3 − 19.5) (*n* = 21)	0.055	0.631	0.553	0.939
sFlt1/s-PlGF								
I trimester	41.3 (37.6 − 45.4) (*n* = 104)	45.4 (39.9 − 51.6) (*n* = 55)	34.1 (30.8 − 37.6) (*n* = 94)	34.2 (30.4 − 38.4) (*n* = 66)	0.396	0.122	0.423	0.410
III trimester	138.9 (114.0 − 169.2) (*n* = 87)	105.2 (81.5 − 135.8) (*n* = 52)	30.0 (20.9 − 43.0) (*n* = 26)	26.5 (17.7 − 39.6) (*n* = 21)	0.210	0.834	0.630	0.324

Data are presented as median (range).

^a^Exercise 2 − 3 times/week or more. ^b^Exercise less than 2 − 3 times/week. ^c^Adjusted for pre-pregnancy BMI, parity, mother’s age at birth and smoking status during pregnancy.

## 4. Discussion

We found no differences in the physical activity and exercise habits between the PE and non-PE women in the FINNPEC cohort. A minority of the women, 43.4% of PE women and 42.4% of non-PE women, were classified as physically active. Further, we found no associations between physical activity in PE or non-PE women and the maternal serum concentrations of sFlt-1, PlGF and sEng and sFlt-1/PlGF ratio.

### 4.1. Effects of physical activity on PE

There is literature in support of a protective role of physical activity on the incidence of PE, but the data are conflicting. In a systematic review and meta-analysis from 2018, a sensitivity analysis of 16 randomized controlled trials (RCTs) showed that prenatal exercise interventions had reduced the odds of developing PE by 41% [4]. Moreover, some earlier systematic reviews and meta-analyses have reported that prenatal physical activity is related to lower incidence of PE [5–7]. Yet, some recent systematic reviews and meta-analyses have found no association between PE and physical activity during pregnancy [8–10, 23, 24]. In a recent systematic review and meta-analysis from 2022, Danielli et al. showed that supervised exercise during pregnancy reduced the risk of developing hypertensive disorders of pregnancy but not independently the risk of PE [24]. An umbrella review of RCTs and updated meta-analysis from 2023 also supports the understanding that exercise during pregnancy does not reduce the incidence of PE [10]. Moreover, the Cochrane review in 2015, evaluating the effectiveness of exercise interventions for preventing excessive weight gain during pregnancy and associated pregnancy complications, did not find any association between PE and exercise interventions [25]. The inconsistency of the study results concerning the association of PE and prenatal exercise may be partly due to the different methods used in the studies to assess physical activity. Moreover, differences in the characteristics of pregnant women and insufficient statistical powers may also be behind the mixed results of the studies [10, 23].

We found no differences in the reported physical activity and exercise habits between the PE and non-PE women. Self-reported information gained through questionnaires is prone to a recall bias, which might have affected our results. Further, the women reported about their average exercise habits during pregnancy, which may have varied from their exercise habits before pregnancy. In their meta-analysis, Davenport et al. stated that to achieve at least a 25% reduction in the odds of developing PE, pregnant women must accumulate at least 140 min of moderate-intensity exercise (brisk walking, water aerobics, stationary cycling or resistance training)/week [4]. Our questionnaire data on physical activity did not allow us to evaluate the amount or intensity of weekly exercise comparably.

### 4.2. Effects of physical activity and PE on angiogenic markers

Increased plasma sFlt-1 levels after acute exercise have been observed in nonpregnant women [15, 26] and in men [27, 28] in a few small studies. The effect of regular exercise has been studied even less. Weissgerber et al. (2010) demonstrated that regular exercise was not associated with sFlt-1, sEng, PlGF or vascular endothelial growth factor (VEGF) concentrations in nonpregnant women [15].

There is very limited data on the association of angiogenic markers and exercise in pregnant women. To our knowledge, this is the first study to investigate the association of physical activity in pregnancy and concentrations of angiogenic markers comparing pregnant PE women with non-PE women. In the current study, we showed that physical activity did not affect the concentrations of angiogenic markers in either PE or non-PE women. Previously, angiogenic markers have been shown to be expressed differently in pregnant women who exercise regularly than in those who do not [15]. Contrary to our results, a cross-sectional study including 25 pregnant women reported higher serum PlGF and lower sFlt-1 and sEng concentrations in the third trimester in physically active pregnant women [15]. Similar to our study, Weissgerber et al. [15] used self-reported questionnaires to define the exercise habits of participants. Yet, the definitions of physically active differed: ours was exercising 2 − 3 times/week or more or exercising ≥ 2–3 times/week, ≥ 30 min at a time with at least moderate intensity, whereas Weissgerber et al. defined it as ‘exercising for at least 3 h/week at an intensity that is sufficient to cause sweating’. Further, the sample size in the Weissgerber et al. study was small compared to the present study.

Previously, rodent studies have investigated the association of exercise training and angiogenic markers in PE. A rat model of hypertension during PE and a rat model of PE superimposed on chronic hypertension showed that exercise training in pregnancy decreased sFlt-1 levels [16, 17]. Contrary to these results, we found no association between physical activity and angiogenic markers in PE women. Gilbert et al. showed that exercise training before and during pregnancy stimulates a pro-angiogenic state/lowers sFlt-1 and increases vascular endothelial growth factor (VEGF) levels in rats [16]. They stated that it remains unclear whether regular exercise training prior to pregnancy was important to their observations. Both pregnancy and exercise can be considerable physiological stressors. Pre-pregnancy exercise status was hypothesized to play a role in whether or not exercise during pregnancy is effective and safe as a preventive measure for increased blood pressure in pregnancy [16]. Our study did not include physical activity before pregnancy, which might have influenced the results.

Enhanced placental growth and vascularity, reduced oxidative stress, reduced inflammation and correction of disease-related endothelial dysfunction have been proposed as the mechanisms behind the protective effect of exercise on developing PE [11]. Further, exercise during pregnancy may promote a pro-angiogenic state [15, 29]. In PE, circulating maternal serum levels of s-Flt-1 and sEng and sFlt-1/PlGF ratio are increased and levels of PlGF are decreased [13, 14]. Equally, in the FINNPEC cohort, the serum concentrations of PlGF and endoglin and the sFlt-1/PlGF ratio were increased in PE women as compared to non-PE women and the serum concentrations of sFlt-1 were increased only at the second/third trimester in PE women [30].

The main strength of this study is that it uses a nationwide, population-based cohort with detailed clinical information from medical records. Self-reported information obtained through questionnaires is prone to recall bias, which can be considered as a limitation. More objective measuring tools, such as pedometers or activity trackers, would be useful in future studies. The inter-individual variations in the serum concentrations of the angiogenic markers were relatively large and the sample size was small, especially when further dividing the study population into subcategories according to exercise status. Moreover, there was a much smaller number of second/third trimester serum samples available from the non-PE women (*n* = 47) compared with PE women (*n* = 139). We acknowledge the need for conducting additional research encompassing larger sample sizes.

## 5. Conclusions

In summary, maternal physical activity during pregnancy was not associated with PE in the FINNPEC cohort. In addition, physical activity of pregnant women with and without PE was not associated with the maternal serum concentrations of angiogenic markers sFlt-1, PlGF and sEng and sFlt-1/PlGF ratio.

## Supplementary Material

Supplemental Material

## Data Availability

The authors confirm that some access restrictions apply to the data. The researchers interested in using the data must obtain approval from the FINNPEC Board (steering committee). The researchers using the data are required to follow the terms of a number of clauses designed to ensure the protection of privacy and compliance with relevant Finnish laws. Data requests may be subject to further review by the Ethics Committee and may also be subject to individual participant consent.

## The Finnish Genetics of Pre-eclampsia Consortium (FINNPEC) core investigator group

Hannele Laivuori, principal investigator, Medical and Clinical Genetics, University of Helsinki and Helsinki University Hospital, Helsinki, Finland; Institute for Molecular Medicine Finland, Helsinki Institute of Life Science, University of Helsinki, Helsinki, Finland; Department of Obstetrics and Gynecology, Tampere University Hospital and Tampere University, Faculty of Medicine and Health Technology, Center for Child, Adolescent, and Maternal Health, Tampere, Finland.

Seppo Heinonen, Obstetrics and Gynecology, University of Helsinki and Helsinki University Hospital, Helsinki, Finland.

Eero Kajantie, PEDEGO Research Unit, Medical Research Center Oulu, Oulu University Hospital and University of Oulu, Oulu, Finland; Public Health Promotion Unit, National Institute for Health and Welfare, Helsinki and Oulu, Finland; Children’s Hospital, University of Helsinki and Helsinki University Hospital, Helsinki, Finland; Department of Clinical and Molecular Medicine, Norwegian University of Health and Technology, Trondheim, Norway.

Juha Kere, Department of Biosciences and Nutrition, Karolinska Institutet, Huddinge, Sweden; Folkhälsan Research Center and Stem Cells and Metabolism Research Program, University of Helsinki, Helsinki, Finland.

Katja Kivinen, Institute for Molecular Medicine Finland, Helsinki Institute of Life Science, University of Helsinki, Helsinki, Finland.

Anneli Pouta, Department of Government Services, National Institute for Health and Welfare, Helsinki, Finland.

## Abbreviations

BMI: Body mass index
FINNPEC: Finnish Genetics of Pre-eclampsia Consortium
PE: Preeclampsia
PlGF: Placental growth factor
RCT: Randomized controlled trial
sFlt-1: Soluble fms-like tyrosine kinase 1
sEng: Soluble endoglin
