# Supplementary material for: Impact of physical activity on preeclampsia and angiogenic markers in the Finnish Genetics of Pre-eclampsia Consortium (FINNPEC) cohort
Source: Ann Med. 2024 Mar 11;56(1):2325480. doi: 10.1080/07853890.2024.2325480 (PMC10930136; doi:10.1080/07853890.2024.2325480)
Supplement: Supplemental Material [file IANN_A_2325480_SM2852.docx]

**Supplementary Table 1. Maternal and perinatal characteristics in the subsets of women with I and II/III trimester serum samples in the FINNPEC.**

|  | **I trimester serum samples** | | | **II/III trimester serum samples** | | |
| --- | --- | --- | --- | --- | --- | --- |
|  | **PE (n=160)** | **Non-PE (n=160)** | ***p*** | **PE (n=139*)*** | **Non-PE (n=47)** | ***p*** |
| Age at delivery (years) | 30.3 ± 4.9 ^a^ | 30.7 ± 4.4 ^a^ | 0.182 | 31.4 ± 5.5 ^a^ | 31.0 ± 5.0 ^a^ | 0.262 |
| Nulliparous | 123 (76.9%) | 95 (59.4%) | **<0.001** | 104 (74.8%) | 30 (63.8%) | 0.147 |
| BMI, kg/m^2^ (self-reported, pre-pregnancy) | 23.7 (17.4−45.1) | 23.3 (18.2­−41.3) | 0.233 | 23.8 (18.2−45.9) | 23.6 (18.9−32.6) | 0.806 |
| Smoking before pregnancy | 39 (24.5%) (n=159) | 34 (21.3%) | 0.486 | 30 (21.6%) | 14 (29.8%) | 0.253 |
| Smoking during pregnancy | 14 (8.8%) (n=159) | 10 (6.3%) | 0.387 | 9 (6.5%) | 4 (8.5%) | 0.636 |
| Highest systolic blood pressure (mmHg) | 165 (130−230) | 126 (102−186) | **<0.001** | 168 (132−207) | 129 (105−190) | **<0.001** |
| Highest diastolic blood pressure (mmHg) | 109 (90−173) | 84 (65−119) | **<0.001** | 111 (97−173) | 86 (67−130) | **<0.001** |
| Highest systolic blood pressure at first antenatal visit (mmHg) | 122 (95−165) (n=158) | 115 (90−153) | **<0.001** | 125 (95−165) | 118 (99−151) | **0.007** |
| Highest diastolic blood pressure at first antenatal visit (mmHg) | 78 (53−104) (n=158) | 71 (50−102) | **<0.001** | 78 (52−100) | 74 (55−99) | **0.003** |
| Chronic hypertension ^b^ | 27 (16.9%) | 6 (3.8%) | **<0.001** | 29 (20.9%) | 5 (10.6%) | 0.117 |
| Gestational hypertension ^c^ | - | 11 (6.9%) |  | - | 7 (14.9%) |  |
| Pre-gestational diabetes | 8 (5.1%) (n=156) | 0 (n=155) | **0.004** | 7 (5.3%) (n=133) | 0 (n=45) | 0.116 |
| Gestational diabetes | 18 (11.3%) | 13 (8.1%) | 0.345 | 20 (14.4%) | 7 (14.9%) | 0.932 |
| Proteinuria without any other signs of PE | - | 4 (2.5%) |  | - | - |  |
| Early onset PE ^d^ | 35 (21.9%) | - |  | 47 (33.8%) | - |  |
| HELLP ^e^ syndrome | 6 (3.8%) | - |  | 10 (7.2%) | - |  |
| Eclampsia ^f^ | 1 (0.6%) | - |  | 1 (0.7%) | - |  |
| Placental insufficiency ^g^ | 14 (9.4%) | 12 (7.5%) | 0.546 | 17 (12.2%) | 10 (21.3%) | 0.128 |
| Thrombocytopenia ^h^ | 39 (33.9%) (n=115) | 9 (9.2%) (n=98) | **<0.001** | 36 (35.6%) (n=101) | 4 (11.4%) (n=35) | **0.007** |
| Elevated ALT ^i^ | 41 (37.6%) (n=198) | 4 (18.2% )(n=22) | 0.08 | 41 (42.7%) (n=96) | 3 (23.1%) (n=13) | 0.176 |
| Mode of delivery |  |  | **<0.001** |  |  | 0.617 |
| Vaginal | 97 (60.6%) | 129 (80.6%) |  | 74 (53.2%) | 27 (57.4%) |  |
| Cesarean section | 63 (39.4%) | 31 (19.4%) |  | 65 (46.8%) | 20 (42.6%) |  |
| Gestational weeks at delivery | 38 (24−42) | 40 (23−42) | **<0.001** | 37 (24−42) | 39 (23−42) | **<0.001** |
| Birth weight, g | 2903 (310−4600) | 3530 (330−4810) | **<0.001** | 2740 (310−4840) | 3504 (330−4565) | **<0.001** |
| Small for gestational age | 26 (16.3%) | 13 (8.1%) | **0.026** | 33 (23.7%) | 10 (21.3%) | 0.729 |

Data are presented as median (range) or percentages.

^a^ Mean ± SD

^b^ Systolic blood pressure ≥ 140 mmHg and/or diastolic blood pressure ≥ 90 mmHg detected before 20 weeks of gestation

^c^ Blood pressure ≥ 140/90, no proteinuria

^d^ PE diagnosed before 34+0 gestational weeks

^e^ Hemolysis, elevated liver enzymes and low platelet count

^f^ New-onset tonic-clonic, focal, or multifocal seizures in a pregnant/postpartum woman in the absence of other causative conditions

^g^ Umbilical artery pulsatility index > +2 SD or umbilical artery resistance index > +2 SD

^h^ Platelet count < 150 x 10^9^/litre

^i^ Alanine aminotransferase > 35 U/litre

Bold text shows *p* values < 0.05

() Number of available information sources if not from all

**Supplementary Table 2. Maternal and perinatal characteristics of the FINNPEC women.**

|  | **PE (n=708)** | **Non-PE (n=724)** | ***p* value** |
| --- | --- | --- | --- |
| Age at delivery, years | 29.9 ± 5.6) ^a^ (n=707) | 29.9 ± 5.1 ^a^ (n=721) | 0.887 |
| Nulliparous | 535 (75.7%) (n=707) | 404 (56.0%) (n=721) | **<0.001** |
| BMI, kg/m^2^ (self-reported, pre-pregnancy) | 24.0 (16.2−47.3) (n=707) | 23.1 (17.0−47.4) (n=721) | **<0.001** |
| Smoking before pregnancy | 208 (29.9%) (n=696) | 209 (29.5%) (n=708) | 0.881 |
| Smoking during pregnancy | 63 (9.0%) (n=700) | 85 (11.8%) (n=720) | 0.084 |
| Highest systolic blood pressure (mmHg) | 164 (118−239) (n=707) | 128 (100−214) (n=721) | **<0.001** |
| Highest diastolic blood pressure (mmHg) | 108 (88−173) (n=707) | 84 (62−134) (n=721) | **<0.001** |
| Highest systolic blood pressure at first antenatal visit (mmHg) | 124 (90−194) (n=689) | 118 (87−190) (n=701) | **<0.001** |
| Highest diastolic blood pressure at first antenatal visit (mmHg) | 78 (52−116) (n=689) | 73 (50−124) (n=701) | **<0.001** |
| Chronic hypertension | 127 (17.9%) | 43 (5.9%) | **<0.001** |
| Gestational hypertension | - | 70 (9.7%) |  |
| Pre-gestational diabetes | 24 (3.6%) (n=667) | 9 (1.3%) (n=693) | **0.006** |
| Gestational diabetes | 114 (16.1%) | 63 (8.7%) | **<0.001** |
| Proteinuria without any other signs of PE | - | 11 (1.5%) |  |
| Early onset PE ^d^ | 157 (22.2%) | - |  |
| HELLP ^e^ syndrome | 30 (4.2%) | - |  |
| Eclampsia ^f^ | 3 (0.4%) | - |  |
| Placental insufficiency ^g^ | 67 (9.5%) | 24 (3.3%) | **<0.001** |
| Thrombocytopenia ^h^ | 143 (27.0%) (n=530) | 28 (6.5%) ((n=434) | **<0.001** |
| Elevated ALT ^i^ | 169 (32.7%) (n=517) | 20 (14.6%) (n=137) | **<0.001** |
| Mode of delivery | (n=707) | (n=723) | **<0.001** |
| Vaginal | 426 (60.3%) | 614 (84.9%) |  |
| Caesarean section | 281 (39.7%) | 109 (15.1%) |  |
| Gestational weeks at delivery | 38 (24-42) (n=707) | 40 (23-43) (n=723) | **<0.001** |
| Birth weight of infant, g | 2910 (310−4840)(n=707) | 3550 (330−5350) (n=723) | **<0.001** |
| Small for gestational age infant | 132 (18.6%) | 36 (5.0%) | **<0.001** |

Data are presented as median (range) or percentages.

^a^ Mean ± SD

^b^ Systolic blood pressure ≥ 140 mmHg and/or diastolic blood pressure ≥ 90 mmHg detected before 20 weeks of gestation

^c^ Blood pressure ≥ 140/90, no proteinuria

^d^ PE diagnosed before 34+0 gestational weeks

^e^ Hemolysis, elevated liver enzymes and low platelet count

^f^ New-onset tonic-clonic, focal, or multifocal seizures in a pregnant/postpartum woman in the absence of other causative conditions

^g^ Umbilical artery pulsatility index > +2 SD or umbilical artery resistance index > +2 SD

^h^ Platelet count < 150 x 10^9^/litre

^i^ Alanine aminotransferase > 35 U/litre

Bold text shows *p* values < 0.05

() Number of available information sources if not from all
